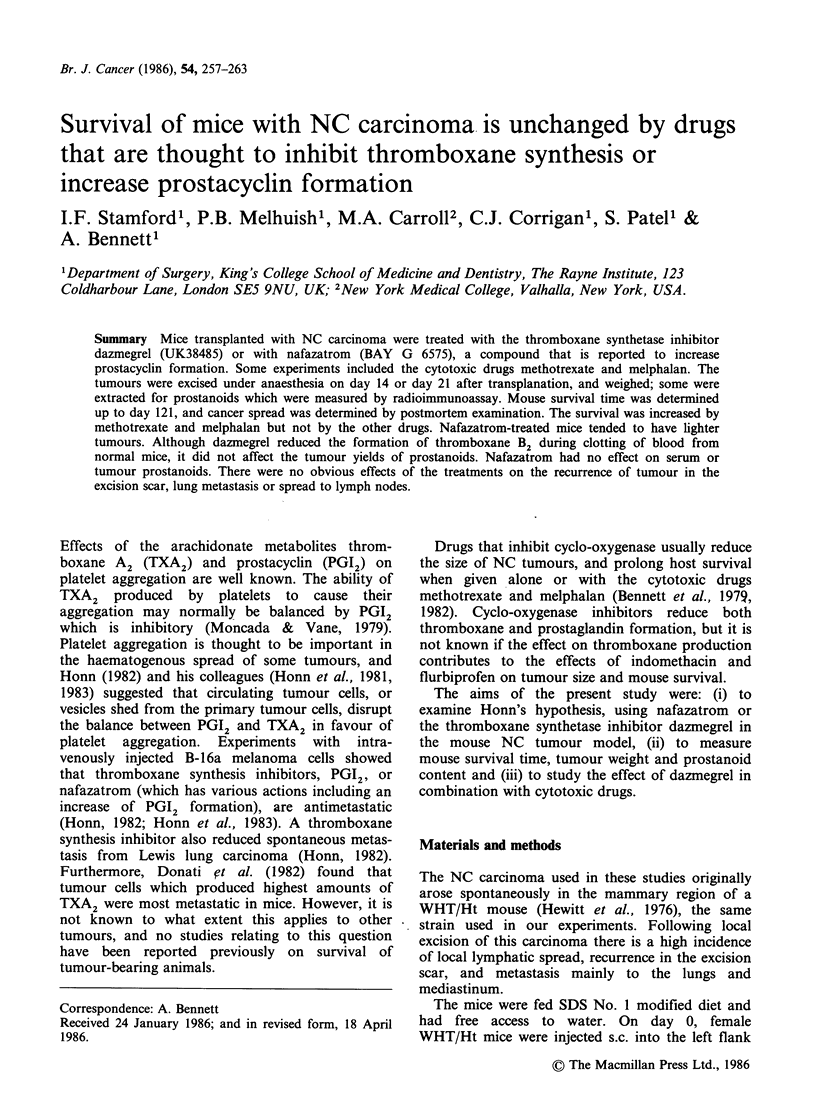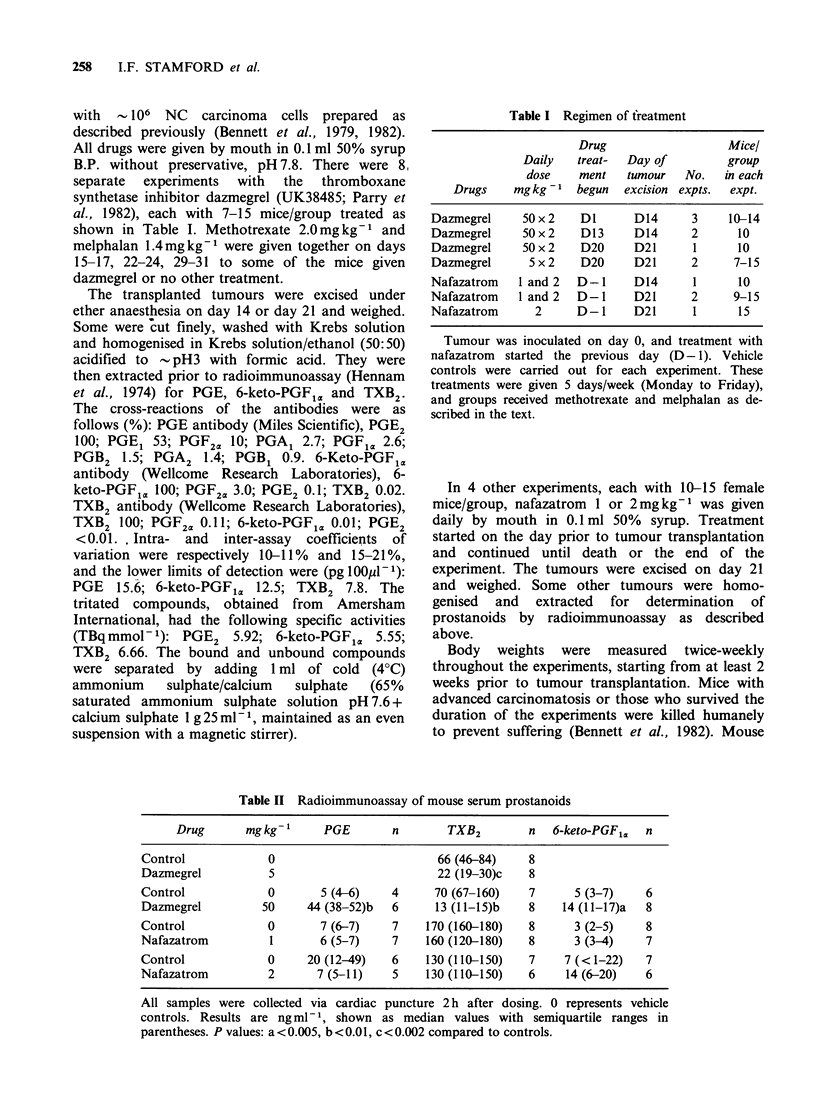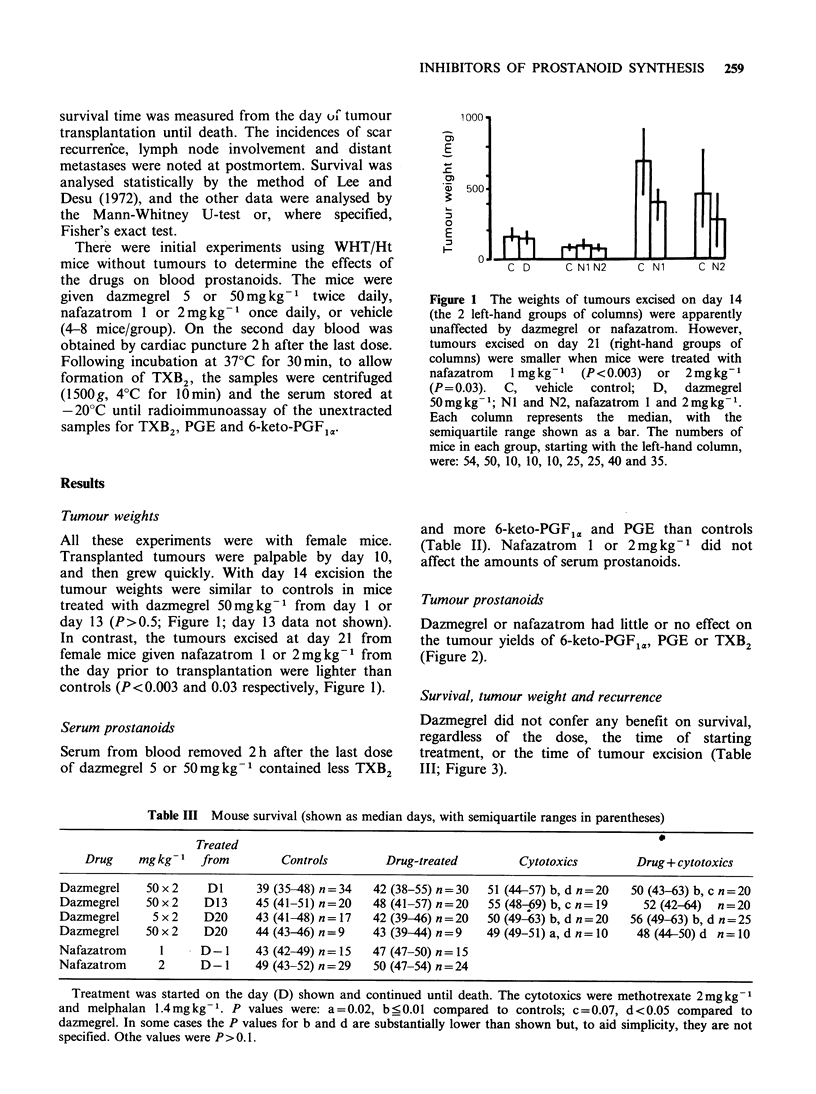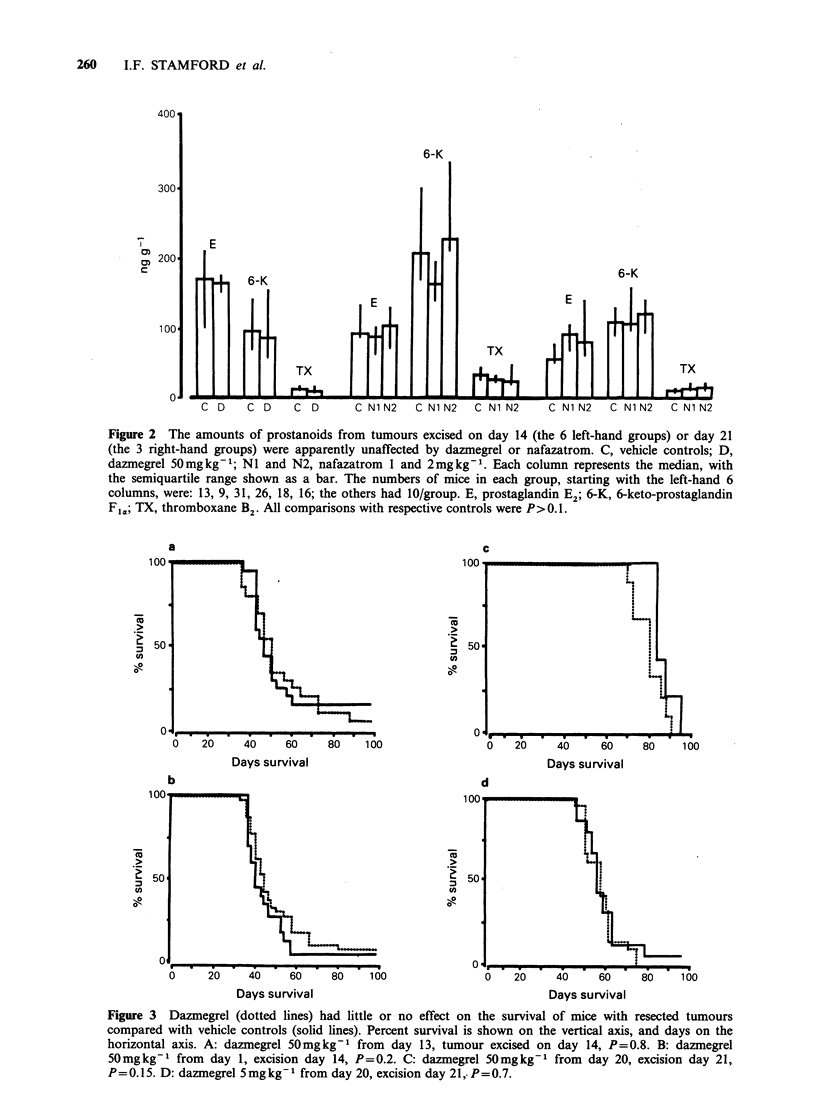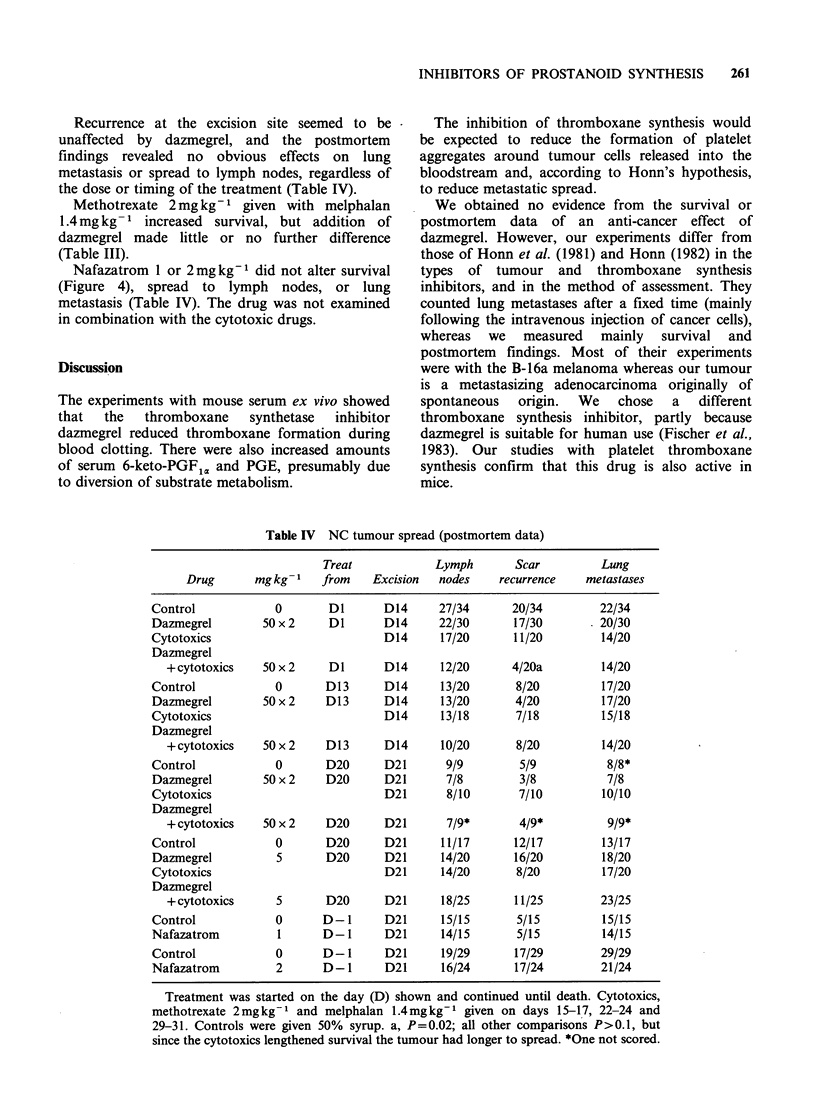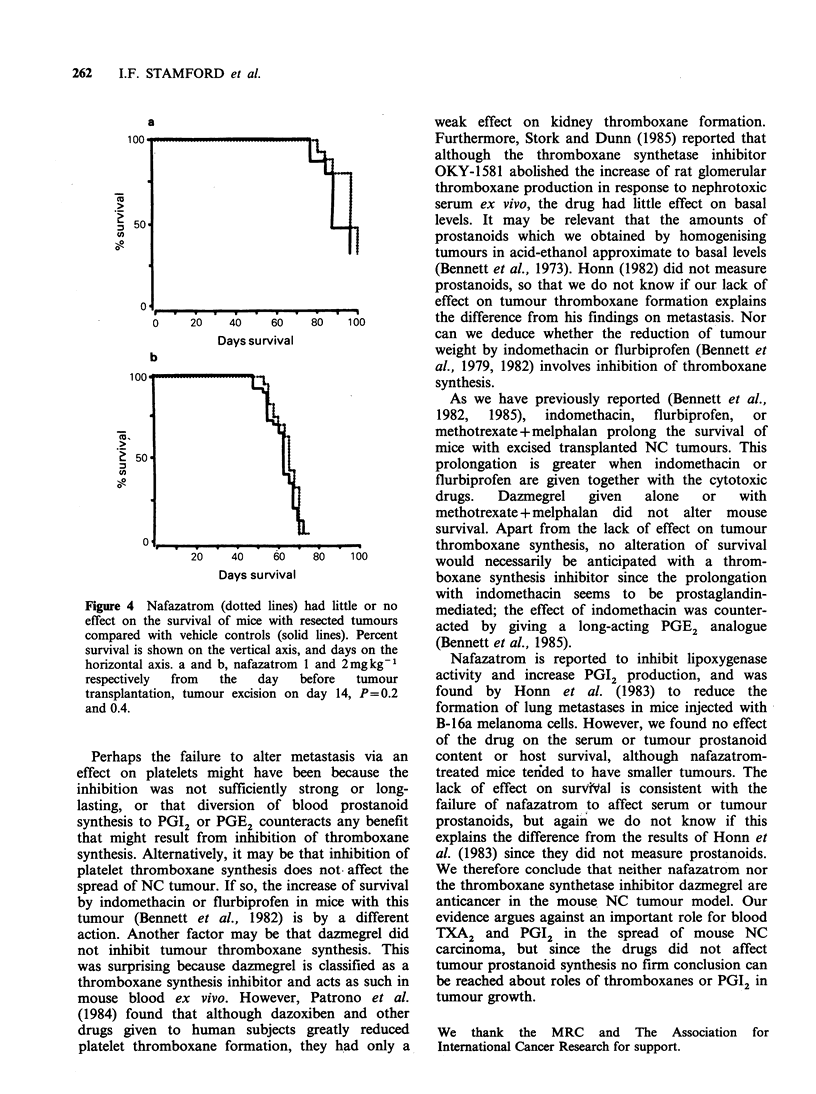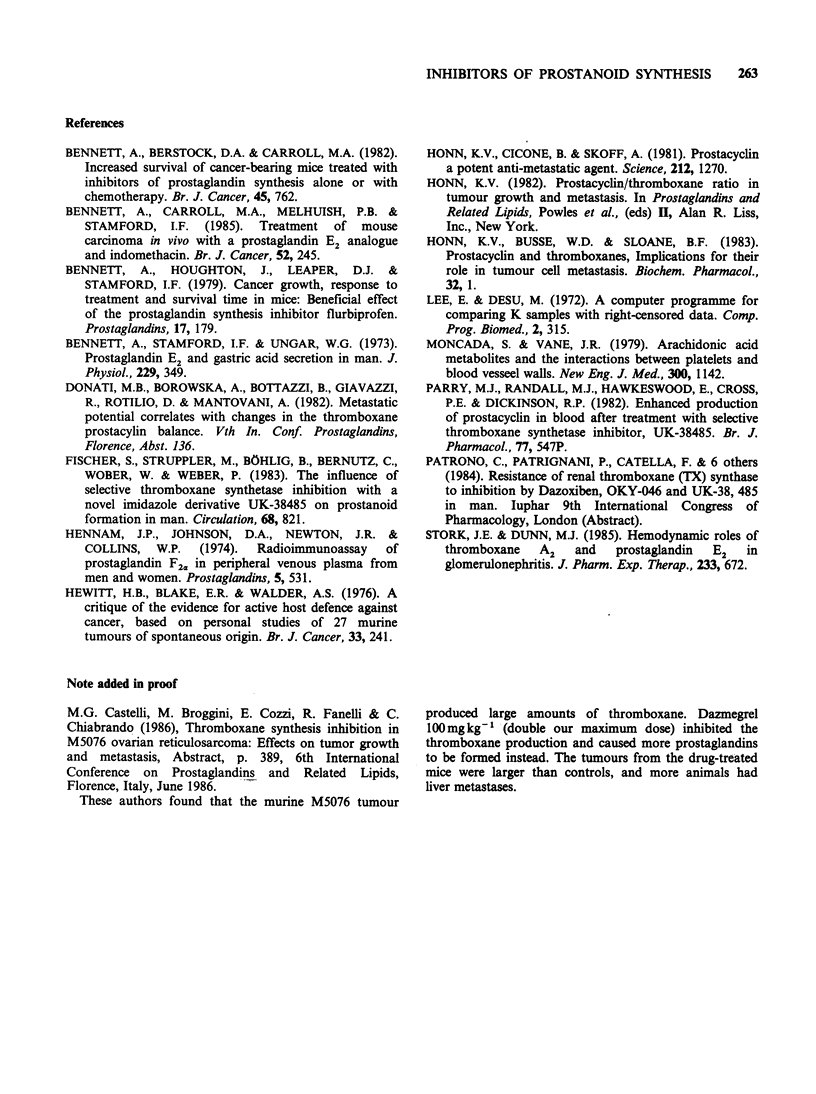# Survival of mice with NC carcinoma is unchanged by drugs that are thought to inhibit thromboxane synthesis or increase prostacyclin formation.

**DOI:** 10.1038/bjc.1986.171

**Published:** 1986-08

**Authors:** I. F. Stamford, P. B. Melhuish, M. A. Carroll, C. J. Corrigan, S. Patel, A. Bennett

## Abstract

Mice transplanted with NC carcinoma were treated with the thromboxane synthetase inhibitor dazmegrel (UK38485) or with nafazatrom (BAY G 6575), a compound that is reported to increase prostacyclin formation. Some experiments included the cytotoxic drugs methotrexate and melphalan. The tumours were excised under anaesthesia on day 14 or day 21 after transplantation, and weighed; some were extracted for prostanoids which were measured by radioimmunoassay. Mouse survival time was determined up to day 121, and cancer spread was determined by postmortem examination. The survival was increased by methotrexate and melphalan but not by the other drugs. Nafazatrom-treated mice tended to have lighter tumours. Although dazmegrel reduced the formation of thromboxane B2 during clotting of blood from normal mice, it did not affect the tumour yields of prostanoids. Nafazatrom had no effect on serum or tumour prostanoids. There were no obvious effects of the treatments on the recurrence of tumour in the excision scar, lung metastasis or spread to lymph nodes.


*Br. J. Cancer* (1986), **54**, 257–263

### Survival of mice with NC carcinoma is unchanged by drugs that are thought to inhibit thromboxane synthesis or increase prostacyclin formation

I.F. Stamford[1], P.B. Melhuish[1], M.A. Carroll[2], C.J. Corrigan[1], S. Patel[1] & A. Bennett[1]

[1]*Department of Surgery, King's College School of Medicine and Dentistry, The Rayne Institute, 123 Coldharbour Lane, London SE5 9NU, UK;* [2]*New York Medical College, Valhalla, New York, USA.*

**Summary** Mice transplanted with NC carcinoma were treated with the thromboxane synthetase inhibitor dazmegrel (UK38485) or with nafazatrom (BAY G 6575), a compound that is reported to increase prostacyclin formation. Some experiments included the cytotoxic drugs methotrexate and melphalan. The tumours were excised under anaesthesia on day 14 or day 21 after transplanation, and weighed; some were extracted for prostanoids which were measured by radioimmunoassay. Mouse survival time was determined up to day 121, and cancer spread was determined by postmortem examination. The survival was increased by methotrexate and melphalan but not by the other drugs. Nafazatrom-treated mice tended to have lighter tumours. Although dazmegrel reduced the formation of thromboxane $B_2$ during clotting of blood from normal mice, it did not affect the tumour yields of prostanoids. Nafazatrom had no effect on serum or tumour prostanoids. There were no obvious effects of the treatments on the recurrence of tumour in the excision scar, lung metastasis or spread to lymph nodes.

Effects of the arachidonate metabolites thromboxane $A_2$ ($TXA_2$) and prostacyclin ($PGI_2$) on platelet aggregation are well known. The ability of $TXA_2$ produced by platelets to cause their aggregation may normally be balanced by $PGI_2$ which is inhibitory (Moncada & Vane, 1979). Platelet aggregation is thought to be important in the haematogenous spread of some tumours, and Honn (1982) and his colleagues (Honn *et al.*, 1981, 1983) suggested that circulating tumour cells, or vesicles shed from the primary tumour cells, disrupt the balance between $PGI_2$ and $TXA_2$ in favour of platelet aggregation. Experiments with intravenously injected B-16a melanoma cells showed that thromboxane synthesis inhibitors, $PGI_2$, or nafazatrom (which has various actions including an increase of $PGI_2$ formation), are antimetastatic (Honn, 1982; Honn *et al.*, 1983). A thromboxane synthesis inhibitor also reduced spontaneous metastasis from Lewis lung carcinoma (Honn, 1982). Furthermore, Donati *et al.* (1982) found that tumour cells which produced highest amounts of $TXA_2$ were most metastatic in mice. However, it is not known to what extent this applies to other tumours, and no studies relating to this question have been reported previously on survival of tumour-bearing animals.

Correspondence: A. Bennett
Received 24 January 1986; and in revised form, 18 April 1986.

Drugs that inhibit cyclo-oxygenase usually reduce the size of NC tumours, and prolong host survival when given alone or with the cytotoxic drugs methotrexate and melphalan (Bennett *et al.*, 1979, 1982). Cyclo-oxygenase inhibitors reduce both thromboxane and prostaglandin formation, but it is not known if the effect on thromboxane production contributes to the effects of indomethacin and flurbiprofen on tumour size and mouse survival.

The aims of the present study were: (i) to examine Honn's hypothesis, using nafazatrom or the thromboxane synthetase inhibitor dazmegrel in the mouse NC tumour model, (ii) to measure mouse survival time, tumour weight and prostanoid content and (iii) to study the effect of dazmegrel in combination with cytotoxic drugs.

### Materials and methods

The NC carcinoma used in these studies originally arose spontaneously in the mammary region of a WHT/Ht mouse (Hewitt *et al.*, 1976), the same strain used in our experiments. Following local excision of this carcinoma there is a high incidence of local lymphatic spread, recurrence in the excision scar, and metastasis mainly to the lungs and mediastinum.

The mice were fed SDS No. 1 modified diet and had free access to water. On day 0, female WHT/Ht mice were injected s.c. into the left flank

© The Macmillan Press Ltd., 1986

with $\sim 10^6$ NC carcinoma cells prepared as described previously (Bennett *et al.*, 1979, 1982). All drugs were given by mouth in 0.1 ml 50% syrup B.P. without preservative, pH 7.8. There were 8 separate experiments with the thromboxane synthetase inhibitor dazmegrel (UK38485; Parry *et al.*, 1982), each with 7–15 mice/group treated as shown in Table I. Methotrexate $2.0\,mg\,kg^{-1}$ and melphalan $1.4\,mg\,kg^{-1}$ were given together on days 15–17, 22–24, 29–31 to some of the mice given dazmegrel or no other treatment.

The transplanted tumours were excised under ether anaesthesia on day 14 or day 21 and weighed. Some were cut finely, washed with Krebs solution and homogenised in Krebs solution/ethanol (50:50) acidified to ~pH3 with formic acid. They were then extracted prior to radioimmunoassay (Hennam *et al.*, 1974) for PGE, 6-keto-$PGF_{1\alpha}$ and $TXB_2$. The cross-reactions of the antibodies were as follows (%): PGE antibody (Miles Scientific), $PGE_2$ 100; $PGE_1$ 53; $PGF_{2\alpha}$ 10; $PGA_1$ 2.7; $PGF_{1\alpha}$ 2.6; $PGB_2$ 1.5; $PGA_2$ 1.4; $PGB_1$ 0.9. 6-Keto-$PGF_{1\alpha}$ antibody (Wellcome Research Laboratories), 6-keto-$PGF_{1\alpha}$ 100; $PGF_{2\alpha}$ 3.0; $PGE_2$ 0.1; $TXB_2$ 0.02. $TXB_2$ antibody (Wellcome Research Laboratories), $TXB_2$ 100; $PGF_{2\alpha}$ 0.11; 6-keto-$PGF_{1\alpha}$ 0.01; $PGE_2$ <0.01. Intra- and inter-assay coefficients of variation were respectively 10–11% and 15–21%, and the lower limits of detection were ($pg\,100\mu l^{-1}$): PGE 15.6; 6-keto-$PGF_{1\alpha}$ 12.5; $TXB_2$ 7.8. The tritated compounds, obtained from Amersham International, had the following specific activities ($TBq\,mmol^{-1}$): $PGE_2$ 5.92; 6-keto-$PGF_{1\alpha}$ 5.55; $TXB_2$ 6.66. The bound and unbound compounds were separated by adding 1 ml of cold (4°C) ammonium sulphate/calcium sulphate (65% saturated ammonium sulphate solution pH 7.6 + calcium sulphate $1\,g\,25\,ml^{-1}$, maintained as an even suspension with a magnetic stirrer).

**Table I** Regimen of treatment

| *Drugs* | *Daily dose mg kg$^{-1}$* | *Drug treat-ment begun* | *Day of tumour excision* | *No. expts.* | *Mice/group in each expt.* |
|---|---|---|---|---|---|
| Dazmegrel | 50 × 2 | D1 | D14 | 3 | 10–14 |
| Dazmegrel | 50 × 2 | D13 | D14 | 2 | 10 |
| Dazmegrel | 50 × 2 | D20 | D21 | 1 | 10 |
| Dazmegrel | 5 × 2 | D20 | D21 | 2 | 7–15 |
| Nafazatrom | 1 and 2 | D − 1 | D14 | 1 | 10 |
| Nafazatrom | 1 and 2 | D − 1 | D21 | 2 | 9–15 |
| Nafazatrom | 2 | D − 1 | D21 | 1 | 15 |

Tumour was inoculated on day 0, and treatment with nafazatrom started the previous day (D − 1). Vehicle controls were carried out for each experiment. These treatments were given 5 days/week (Monday to Friday), and groups received methotrexate and melphalan as described in the text.

In 4 other experiments, each with 10–15 female mice/group, nafazatrom 1 or $2\,mg\,kg^{-1}$ was given daily by mouth in 0.1 ml 50% syrup. Treatment started on the day prior to tumour transplantation and continued until death or the end of the experiment. The tumours were excised on day 21 and weighed. Some other tumours were homogenised and extracted for determination of prostanoids by radioimmunoassay as described above.

Body weights were measured twice-weekly throughout the experiments, starting from at least 2 weeks prior to tumour transplantation. Mice with advanced carcinomatosis or those who survived the duration of the experiments were killed humanely to prevent suffering (Bennett *et al.*, 1982). Mouse

**Table II** Radioimmunoassay of mouse serum prostanoids

| *Drug* | *mg kg$^{-1}$* | *PGE* | *n* | *$TXB_2$* | *n* | *6-keto-$PGF_{1\alpha}$* | *n* |
|---|---|---|---|---|---|---|---|
| Control | 0 | | | 66 (46–84) | 8 | | |
| Dazmegrel | 5 | | | 22 (19–30)c | 8 | | |
| Control | 0 | 5 (4–6) | 4 | 70 (67–160) | 7 | 5 (3–7) | 6 |
| Dazmegrel | 50 | 44 (38–52)b | 6 | 13 (11–15)b | 8 | 14 (11–17)a | 8 |
| Control | 0 | 7 (6–7) | 7 | 170 (160–180) | 8 | 3 (2–5) | 8 |
| Nafazatrom | 1 | 6 (5–7) | 7 | 160 (120–180) | 8 | 3 (3–4) | 7 |
| Control | 0 | 20 (12–49) | 6 | 130 (110–150) | 7 | 7 (<1–22) | 7 |
| Nafazatrom | 2 | 7 (5–11) | 5 | 130 (110–150) | 6 | 14 (6–20) | 6 |

All samples were collected via cardiac puncture 2 h after dosing. 0 represents vehicle controls. Results are $ng\,ml^{-1}$, shown as median values with semiquartile ranges in parentheses. *P* values: a < 0.005, b < 0.01, c < 0.002 compared to controls.

survival time was measured from the day of tumour transplantation until death. The incidences of scar recurrence, lymph node involvement and distant metastases were noted at postmortem. Survival was analysed statistically by the method of Lee and Desu (1972), and the other data were analysed by the Mann-Whitney U-test or, where specified, Fisher's exact test.

There were initial experiments using WHT/Ht mice without tumours to determine the effects of the drugs on blood prostanoids. The mice were given dazmegrel 5 or 50 mg kg$^{-1}$ twice daily, nafazatrom 1 or 2 mg kg$^{-1}$ once daily, or vehicle (4–8 mice/group). On the second day blood was obtained by cardiac puncture 2 h after the last dose. Following incubation at 37°C for 30 min, to allow formation of $TXB_2$, the samples were centrifuged (1500 *g*, 4°C for 10 min) and the serum stored at −20°C until radioimmunoassay of the unextracted samples for $TXB_2$, PGE and 6-keto-$PGF_{1\alpha}$.

**Figure 1** The weights of tumours excised on day 14 (the 2 left-hand groups of columns) were apparently unaffected by dazmegrel or nafazatrom. However, tumours excised on day 21 (right-hand groups of columns) were smaller when mice were treated with nafazatrom 1 mg kg$^{-1}$ ($P<0.003$) or 2 mg kg$^{-1}$ ($P=0.03$). C, vehicle control; D, dazmegrel 50 mg kg$^{-1}$; N1 and N2, nafazatrom 1 and 2 mg kg$^{-1}$. Each column represents the median, with the semiquartile range shown as a bar. The numbers of mice in each group, starting with the left-hand column, were: 54, 50, 10, 10, 10, 25, 25, 40 and 35.

### Results

### *Tumour weights*

All these experiments were with female mice. Transplanted tumours were palpable by day 10, and then grew quickly. With day 14 excision the tumour weights were similar to controls in mice treated with dazmegrel 50 mg kg$^{-1}$ from day 1 or day 13 ($P>0.5$; Figure 1; day 13 data not shown). In contrast, the tumours excised at day 21 from female mice given nafazatrom 1 or 2 mg kg$^{-1}$ from the day prior to transplantation were lighter than controls ($P<0.003$ and 0.03 respectively, Figure 1).

### *Serum prostanoids*

Serum from blood removed 2 h after the last dose of dazmegrel 5 or 50 mg kg$^{-1}$ contained less $TXB_2$ and more 6-keto-$PGF_{1\alpha}$ and PGE than controls (Table II). Nafazatrom 1 or 2 mg kg$^{-1}$ did not affect the amounts of serum prostanoids.

### *Tumour prostanoids*

Dazmegrel or nafazatrom had little or no effect on the tumour yields of 6-keto-$PGF_{1\alpha}$, PGE or $TXB_2$ (Figure 2).

### *Survival, tumour weight and recurrence*

Dazmegrel did not confer any benefit on survival, regardless of the dose, the time of starting treatment, or the time of tumour excision (Table III; Figure 3).

**Table III** Mouse survival (shown as median days, with semiquartile ranges in parentheses)

| *Drug* | *mg kg$^{-1}$* | *Treated from* | *Controls* | *Drug-treated* | *Cytotoxics* | *Drug + cytotoxics* |
|---|---|---|---|---|---|---|
| Dazmegrel | 50 × 2 | D1 | 39 (35–48) $n=34$ | 42 (38–55) $n=30$ | 51 (44–57) b, d $n=20$ | 50 (43–63) b, c $n=20$ |
| Dazmegrel | 50 × 2 | D13 | 45 (41–51) $n=20$ | 48 (41–57) $n=20$ | 55 (48–69) b, c $n=19$ | 52 (42–64) $n=20$ |
| Dazmegrel | 5 × 2 | D20 | 43 (41–48) $n=17$ | 42 (39–46) $n=20$ | 50 (49–63) b, d $n=20$ | 56 (49–63) b, d $n=25$ |
| Dazmegrel | 50 × 2 | D20 | 44 (43–46) $n=9$ | 43 (39–44) $n=9$ | 49 (49–51) a, d $n=10$ | 48 (44–50) d $n=10$ |
| Nafazatrom | 1 | D − 1 | 43 (42–49) $n=15$ | 47 (47–50) $n=15$ | | |
| Nafazatrom | 2 | D − 1 | 49 (43–52) $n=29$ | 50 (47–54) $n=24$ | | |

Treatment was started on the day (D) shown and continued until death. The cytotoxics were methotrexate 2 mg kg$^{-1}$ and melphalan 1.4 mg kg$^{-1}$. *P* values were: a = 0.02, b ≤ 0.01 compared to controls; c = 0.07, d < 0.05 compared to dazmegrel. In some cases the *P* values for b and d are substantially lower than shown but, to aid simplicity, they are not specified. Othe values were $P>0.1$.

**Figure 2** The amounts of prostanoids from tumours excised on day 14 (the 6 left-hand groups) or day 21 (the 3 right-hand groups) were apparently unaffected by dazmegrel or nafazatrom. C, vehicle controls; D, dazmegrel $50 \, mg \, kg^{-1}$; N1 and N2, nafazatrom 1 and $2 \, mg \, kg^{-1}$. Each column represents the median, with the semiquartile range shown as a bar. The numbers of mice in each group, starting with the left-hand 6 columns, were: 13, 9, 31, 26, 18, 16; the others had 10/group. E, prostaglandin $E_2$; 6-K, 6-keto-prostaglandin $F_{1\alpha}$; TX, thromboxane $B_2$. All comparisons with respective controls were $P>0.1$.

**Figure 3** Dazmegrel (dotted lines) had little or no effect on the survival of mice with resected tumours compared with vehicle controls (solid lines). Percent survival is shown on the vertical axis, and days on the horizontal axis. A: dazmegrel $50 \, mg \, kg^{-1}$ from day 13, tumour excised on day 14, $P=0.8$. B: dazmegrel $50 \, mg \, kg^{-1}$ from day 1, excision day 14, $P=0.2$. C: dazmegrel $50 \, mg \, kg^{-1}$ from day 20, excision day 21, $P=0.15$. D: dazmegrel $5 \, mg \, kg^{-1}$ from day 20, excision day 21, $P=0.7$.

Recurrence at the excision site seemed to be unaffected by dazmegrel, and the postmortem findings revealed no obvious effects on lung metastasis or spread to lymph nodes, regardless of the dose or timing of the treatment (Table IV).

Methotrexate $2\,mg\,kg^{-1}$ given with melphalan $1.4\,mg\,kg^{-1}$ increased survival, but addition of dazmegrel made little or no further difference (Table III).

Nafazatrom 1 or $2\,mg\,kg^{-1}$ did not alter survival (Figure 4), spread to lymph nodes, or lung metastasis (Table IV). The drug was not examined in combination with the cytotoxic drugs.

### Discussion

The experiments with mouse serum *ex vivo* showed that the thromboxane synthetase inhibitor dazmegrel reduced thromboxane formation during blood clotting. There were also increased amounts of serum 6-keto-$PGF_{1\alpha}$ and PGE, presumably due to diversion of substrate metabolism.

The inhibition of thromboxane synthesis would be expected to reduce the formation of platelet aggregates around tumour cells released into the bloodstream and, according to Honn's hypothesis, to reduce metastatic spread.

We obtained no evidence from the survival or postmortem data of an anti-cancer effect of dazmegrel. However, our experiments differ from those of Honn *et al.* (1981) and Honn (1982) in the types of tumour and thromboxane synthesis inhibitors, and in the method of assessment. They counted lung metastases after a fixed time (mainly following the intravenous injection of cancer cells), whereas we measured mainly survival and postmortem findings. Most of their experiments were with the B-16a melanoma whereas our tumour is a metastasizing adenocarcinoma originally of spontaneous origin. We chose a different thromboxane synthesis inhibitor, partly because dazmegrel is suitable for human use (Fischer *et al.*, 1983). Our studies with platelet thromboxane synthesis confirm that this drug is also active in mice.

**Table IV** NC tumour spread (postmortem data)

| *Drug* | *mg kg⁻¹* | *Treat from* | *Excision* | *Lymph nodes* | *Scar recurrence* | *Lung metastases* |
|---|---|---|---|---|---|---|
| Control | 0 | D1 | D14 | 27/34 | 20/34 | 22/34 |
| Dazmegrel | 50×2 | D1 | D14 | 22/30 | 17/30 | 20/30 |
| Cytotoxics | | | D14 | 17/20 | 11/20 | 14/20 |
| Dazmegrel +cytotoxics | 50×2 | D1 | D14 | 12/20 | 4/20a | 14/20 |
| Control | 0 | D13 | D14 | 13/20 | 8/20 | 17/20 |
| Dazmegrel | 50×2 | D13 | D14 | 13/20 | 4/20 | 17/20 |
| Cytotoxics | | | D14 | 13/18 | 7/18 | 15/18 |
| Dazmegrel +cytotoxics | 50×2 | D13 | D14 | 10/20 | 8/20 | 14/20 |
| Control | 0 | D20 | D21 | 9/9 | 5/9 | 8/8* |
| Dazmegrel | 50×2 | D20 | D21 | 7/8 | 3/8 | 7/8 |
| Cytotoxics | | | D21 | 8/10 | 7/10 | 10/10 |
| Dazmegrel +cytotoxics | 50×2 | D20 | D21 | 7/9* | 4/9* | 9/9* |
| Control | 0 | D20 | D21 | 11/17 | 12/17 | 13/17 |
| Dazmegrel | 5 | D20 | D21 | 14/20 | 16/20 | 18/20 |
| Cytotoxics | | | D21 | 14/20 | 8/20 | 17/20 |
| Dazmegrel +cytotoxics | 5 | D20 | D21 | 18/25 | 11/25 | 23/25 |
| Control | 0 | D−1 | D21 | 15/15 | 5/15 | 15/15 |
| Nafazatrom | 1 | D−1 | D21 | 14/15 | 5/15 | 14/15 |
| Control | 0 | D−1 | D21 | 19/29 | 17/29 | 29/29 |
| Nafazatrom | 2 | D−1 | D21 | 16/24 | 17/24 | 21/24 |

Treatment was started on the day (D) shown and continued until death. Cytotoxics, methotrexate $2\,mg\,kg^{-1}$ and melphalan $1.4\,mg\,kg^{-1}$ given on days 15–17, 22–24 and 29–31. Controls were given 50% syrup. a, $P=0.02$; all other comparisons $P>0.1$, but since the cytotoxics lengthened survival the tumour had longer to spread. *One not scored.

**Figure 4** Nafazatrom (dotted lines) had little or no effect on the survival of mice with resected tumours compared with vehicle controls (solid lines). Percent survival is shown on the vertical axis, and days on the horizontal axis. a and b, nafazatrom 1 and 2 mg kg$^{-1}$ respectively from the day before tumour transplantation, tumour excision on day 14, $P=0.2$ and 0.4.

Perhaps the failure to alter metastasis via an effect on platelets might have been because the inhibition was not sufficiently strong or long-lasting, or that diversion of blood prostanoid synthesis to $PGI_2$ or $PGE_2$ counteracts any benefit that might result from inhibition of thromboxane synthesis. Alternatively, it may be that inhibition of platelet thromboxane synthesis does not affect the spread of NC tumour. If so, the increase of survival by indomethacin or flurbiprofen in mice with this tumour (Bennett *et al.*, 1982) is by a different action. Another factor may be that dazmegrel did not inhibit tumour thromboxane synthesis. This was surprising because dazmegrel is classified as a thromboxane synthesis inhibitor and acts as such in mouse blood *ex vivo*. However, Patrono *et al.* (1984) found that although dazoxiben and other drugs given to human subjects greatly reduced platelet thromboxane formation, they had only a weak effect on kidney thromboxane formation. Furthermore, Stork and Dunn (1985) reported that although the thromboxane synthetase inhibitor OKY-1581 abolished the increase of rat glomerular thromboxane production in response to nephrotoxic serum *ex vivo*, the drug had little effect on basal levels. It may be relevant that the amounts of prostanoids which we obtained by homogenising tumours in acid-ethanol approximate to basal levels (Bennett *et al.*, 1973). Honn (1982) did not measure prostanoids, so that we do not know if our lack of effect on tumour thromboxane formation explains the difference from his findings on metastasis. Nor can we deduce whether the reduction of tumour weight by indomethacin or flurbiprofen (Bennett *et al.*, 1979, 1982) involves inhibition of thromboxane synthesis.

As we have previously reported (Bennett *et al.*, 1982, 1985), indomethacin, flurbiprofen, or methotrexate + melphalan prolong the survival of mice with excised transplanted NC tumours. This prolongation is greater when indomethacin or flurbiprofen are given together with the cytotoxic drugs. Dazmegrel given alone or with methotrexate + melphalan did not alter mouse survival. Apart from the lack of effect on tumour thromboxane synthesis, no alteration of survival would necessarily be anticipated with a thromboxane synthesis inhibitor since the prolongation with indomethacin seems to be prostaglandin-mediated; the effect of indomethacin was counteracted by giving a long-acting $PGE_2$ analogue (Bennett *et al.*, 1985).

Nafazatrom is reported to inhibit lipoxygenase activity and increase $PGI_2$ production, and was found by Honn *et al.* (1983) to reduce the formation of lung metastases in mice injected with B-16a melanoma cells. However, we found no effect of the drug on the serum or tumour prostanoid content or host survival, although nafazatrom-treated mice tended to have smaller tumours. The lack of effect on survival is consistent with the failure of nafazatrom to affect serum or tumour prostanoids, but again we do not know if this explains the difference from the results of Honn *et al.* (1983) since they did not measure prostanoids. We therefore conclude that neither nafazatrom nor the thromboxane synthetase inhibitor dazmegrel are anticancer in the mouse NC tumour model. Our evidence argues against an important role for blood $TXA_2$ and $PGI_2$ in the spread of mouse NC carcinoma, but since the drugs did not affect tumour prostanoid synthesis no firm conclusion can be reached about roles of thromboxanes or $PGI_2$ in tumour growth.

We thank the MRC and The Association for International Cancer Research for support.

### References

BENNETT, A., BERSTOCK, D.A. & CARROLL, M.A. (1982). Increased survival of cancer-bearing mice treated with inhibitors of prostaglandin synthesis alone or with chemotherapy. *Br. J. Cancer*, **45**, 762.

BENNETT, A., CARROLL, M.A., MELHUISH, P.B. & STAMFORD, I.F. (1985). Treatment of mouse carcinoma *in vivo* with a prostaglandin $E_2$ analogue and indomethacin. *Br. J. Cancer*, **52**, 245.

BENNETT, A., HOUGHTON, J., LEAPER, D.J. & STAMFORD, I.F. (1979). Cancer growth, response to treatment and survival time in mice: Beneficial effect of the prostaglandin synthesis inhibitor flurbiprofen. *Prostaglandins*, **17**, 179.

BENNETT, A., STAMFORD, I.F. & UNGAR, W.G. (1973). Prostaglandin $E_2$ and gastric acid secretion in man. *J. Physiol.*, **229**, 349.

DONATI, M.B., BOROWSKA, A., BOTTAZZI, B., GIAVAZZI, R., ROTILIO, D. & MANTOVANI, A. (1982). Metastatic potential correlates with changes in the thromboxane prostacylin balance. *Vth In. Conf. Prostaglandins, Florence, Abst. 136.*

FISCHER, S., STRUPPLER, M., BÖHLIG, B., BERNUTZ, C., WOBER, W. & WEBER, P. (1983). The influence of selective thromboxane synthetase inhibition with a novel imidazole derivative UK-38485 on prostanoid formation in man. *Circulation*, **68**, 821.

HENNAM, J.P., JOHNSON, D.A., NEWTON, J.R. & COLLINS, W.P. (1974). Radioimmunoassay of prostaglandin $F_{2\alpha}$ in peripheral venous plasma from men and women. *Prostaglandins*, **5**, 531.

HEWITT, H.B., BLAKE, E.R. & WALDER, A.S. (1976). A critique of the evidence for active host defence against cancer, based on personal studies of 27 murine tumours of spontaneous origin. *Br. J. Cancer*, **33**, 241.

HONN, K.V., CICONE, B. & SKOFF, A. (1981). Prostacyclin a potent anti-metastatic agent. *Science*, **212**, 1270.

HONN, K.V. (1982). Prostacyclin/thromboxane ratio in tumour growth and metastasis. In *Prostaglandins and Related Lipids*, Powles *et al.*, (eds) **II**, Alan R. Liss, Inc., New York.

HONN, K.V., BUSSE, W.D. & SLOANE, B.F. (1983). Prostacyclin and thromboxanes, Implications for their role in tumour cell metastasis. *Biochem. Pharmacol.*, **32**, 1.

LEE, E. & DESU, M. (1972). A computer programme for comparing K samples with right-censored data. *Comp. Prog. Biomed.*, **2**, 315.

MONCADA, S. & VANE, J.R. (1979). Arachidonic acid metabolites and the interactions between platelets and blood vesseel walls. *New Eng. J. Med.*, **300**, 1142.

PARRY, M.J., RANDALL, M.J., HAWKESWOOD, E., CROSS, P.E. & DICKINSON, R.P. (1982). Enhanced production of prostacyclin in blood after treatment with selective thromboxane synthetase inhibitor, UK-38485. *Br. J. Pharmacol.*, **77**, 547P.

PATRONO, C., PATRIGNANI, P., CATELLA, F. & 6 others (1984). Resistance of renal thromboxane (TX) synthase to inhibition by Dazoxiben, OKY-046 and UK-38, 485 in man. Iuphar 9th International Congress of Pharmacology, London (Abstract).

STORK, J.E. & DUNN, M.J. (1985). Hemodynamic roles of thromboxane $A_2$ and prostaglandin $E_2$ in glomerulonephritis. *J. Pharm. Exp. Therap.*, **233**, 672.

### Note added in proof

M.G. Castelli, M. Broggini, E. Cozzi, R. Fanelli & C. Chiabrando (1986), Thromboxane synthesis inhibition in M5076 ovarian reticulosarcoma: Effects on tumor growth and metastasis, Abstract, p. 389, 6th International Conference on Prostaglandins and Related Lipids, Florence, Italy, June 1986.

These authors found that the murine M5076 tumour produced large amounts of thromboxane. Dazmegrel $100\,mg\,kg^{-1}$ (double our maximum dose) inhibited the thromboxane production and caused more prostaglandins to be formed instead. The tumours from the drug-treated mice were larger than controls, and more animals had liver metastases.